# Predictors of Recurrence in Patients with Papillary Thyroid Carcinoma: Does Male Sex Matter?

**DOI:** 10.3390/cancers14081896

**Published:** 2022-04-09

**Authors:** Hyeji Kim, Hyungju Kwon, Byung-In Moon

**Affiliations:** Department of Surgery, Ewha Womans University Medical Center, 1071 Anyangcheon-ro, Yangcheon-Gu, Seoul 07985, Korea; rlagpwl1003@gmail.com (H.K.); mbit@ewha.ac.kr (B.-I.M.)

**Keywords:** papillary thyroid carcinoma, male, sex, recurrence

## Abstract

**Simple Summary:**

Men with papillary thyroid carcinoma tend to have more high-risk features for recurrence than women. However, the prognostic impact of sex remains controversial and unestablished. Our study of 1252 patients indicated that men had risk of recurrence comparable to that of women, although male sex was associated with more aggressive disease. Furthermore, we also confirmed that the impact of sex for recurrence was not associated with tumor size or patient age. In conclusion, male sex did not increase the risk of recurrence in patients with papillary thyroid carcinoma. Male patients do not always require aggressive treatment and follow-up approaches.

**Abstract:**

Male patients with papillary thyroid carcinoma (PTC) usually have aggressive clinicopathological features, including large tumor size and lymph node metastasis; however, it is unclear whether male sex increases the risk of recurrence. Here, we evaluated the effect of sex on disease-free survival (DFS) of patients with PTC. Between 2009 and 2016, 1252 patients who underwent total thyroidectomy for PTC were enrolled; 157 (12.5%) were male and 1095 (87.5%) were female. With a mean follow-up of 6.6 years, five-year DFS rates were comparable between male and female patients (94.9% vs. 96.9%; *p* = 0.616) after adjusting for potential confounders. Multivariate Cox regression analysis also demonstrated that male sex was not an independent risk factor for recurrence (HR 1.982, 95% CI 0.831–4.726). Subgroup analyses further indicated that both male and female sex—in terms of their associations with five-year DFS—were comparable with other variables, including age < 55 years (94.5% vs. 97.3%; *p* = 0.520) and tumor size > 1 cm (91.9% vs. 97.0%; *p* = 0.243). In conclusion, male sex was not associated with the risk of recurrence in patients with PTC. Male patients do not always require aggressive treatment and follow-up approaches.

## 1. Introduction

The incidence of thyroid cancer has rapidly risen over the last four decades worldwide [1,2]. There were 586,202 new cases of thyroid cancer worldwide in 2020, accounting for 3.0% of all cancer patients. Papillary thyroid carcinoma (PTC) is the most common type of thyroid malignancy, constituting more than 80% of thyroid cancers. Patients with PTC usually have excellent outcomes with appropriate therapy, while up to 30% of patients present with aggressive disease, including patients with locoregional recurrence and distant metastasis [3,4]. Researchers have tried to differentiate these high-risk patients from the population with favorable prognoses [5,6,7]. Many clinical and pathological factors, including sex, age, tumor size, and lymph node (LN) metastasis, have been thoroughly investigated in efforts to identify predictors of recurrence.

Tumor biology is strongly affected by sexual dimorphism in many cancers, including thyroid cancer [8]. Demographic, clinical, and pathological characteristics of thyroid cancer can vary according to sex. The age-standardized incidence rates per 100,000 men and women were 3.1 and 10.1 in 2020 [1]. The mortality rates associated with thyroid cancer were 0.3 per 100,000 men and 0.5 per 100,000 women. Women have higher incidence and mortality; however, men tend to have more aggressive cancer at diagnosis [9]. In PTC, male sex is associated with larger tumor size, LN metastasis, higher American Joint Committee on Cancer (AJCC) stage, more extensive surgery (including neck dissection), and administration of radioactive iodine [10,11,12].

There is an ongoing debate regarding the prognostic significance of sex in PTC. A Surveillance, Epidemiology, and End Results (SEER) database analysis showed that male sex was an independent indicator of poor prognosis for patients with PTC [12]. A registry study from Canada further suggested that the risk of recurrence in patients with well-differentiated thyroid cancer was higher in men than in women [10]. A meta-analysis in 2014 also indicated that male sex was associated with higher risk of recurrence [13]. In contrast, recent studies with large cohorts have demonstrated that male and female patients have comparable recurrence rates [11,14,15,16,17]. These controversial data have resulted from, at least in part, heterogeneity among study populations and other factors, such as wide variations in operative extent. Here, we evaluated the impact of sex on the recurrence of PTC in a homogeneous cohort.

## 2. Materials and Methods

### 2.1. Study Design

Our institutional review board (Approval No. 2022-04-004) approved this retrospective cohort study and waived the requirement for written informed consent. This study included 1252 consecutive PTC patients who underwent total thyroidectomy from January 2009 through December 2016 at Ewha Womans University Medical Center (Seoul, Korea). All patients underwent neck ultrasonography and computed tomography preoperatively to evaluate LN metastasis. Therapeutic LN dissection in addition to total thyroidectomy was performed for patients with suspicious LN enlargement.

Clinicopathological features, including tumor size, extrathyroidal extension (ETE), multifocality, LN metastasis, resection margin involvement, and coexisting Hashimoto thyroiditis, and data about adjuvant radioiodine treatment were collected. The AJCC 7th edition was used for Tumor-Node-Metastasis (TNM) staging. Follow-up duration and recurrence status data were also recorded and analyzed.

### 2.2. Outcome Measures

Primary outcome measure was 5-year disease-free survival (DFS), which was defined as the percentage of patients who were alive without recurrence 5 years after their initial surgery. Recurrence was defined as newly detected malignant lesions on the operative bed, metastatic LNs, or distant metastasis at least 1 year after the initial surgery, with histopathologic or radiologic confirmation.

### 2.3. Subgroup Analyses

Previous studies have suggested that the impact of sex might vary according to patient age (<55 years vs. ≥55 years) and tumor size (≤1 cm vs. >1 cm). Subgroup analyses were performed, therefore, to evaluate the impact of sex in PTC patients with adjustments for possible confounders.

### 2.4. Statistical Analysis

R 4.1.2 (R Foundation for Statistical Computing, Vienna, Austria) and SPSS Statistics for Windows, version 23.0 (IBM Corp., Armonk, NY, USA) were used for statistical analysis. Continuous data were compared using Student’s *t*-tests. Comparison of dichotomous data were performed using chi-squared tests. DFS was assessed using Kaplan–Meier survival plots and the log-rank test. To adjust possible confounders and minimize selection bias, 1:3 propensity score matching was performed. We selected four factors which could affect recurrence: tumor size, ETE, LN metastasis, and coexisting Hashimoto thyroiditis. Cox proportional hazards model was used to investigate the association between prognostic factors and recurrence. A *p*-value < 0.05 was considered statistically significant.

## 3. Results

### 3.1. Patient Demographics and Tumor Characteristics

Table 1 summarizes the clinicopathological characteristics of the included patients. Of the 1252 patients included, 157 (12.5%) were men and 1095 (87.5) were women. Mean age at the time of surgery was 47.5 ± 11.2 years. Mean follow-up period was 6.6 ± 3.1 years), and 27 (2.2%) patients developed recurrences.

**Figure 1:**
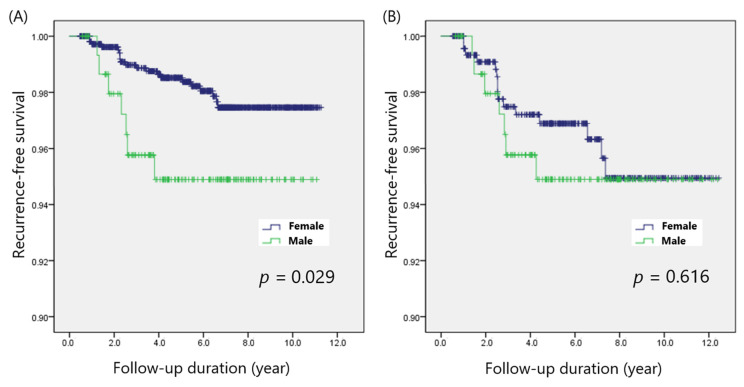
Disease-free survival according to sex, (**A**) before and (**B**) after propensity score matching.

Male patients had larger tumor size (1.3 ± 0.9 cm vs. 1.0 ± 0.6 cm; *p* < 0.001), more ETE (1.9% vs. 0.3%; *p* = 0.018), more LN metastasis (16.6% vs. 8.7%; *p* = 0.001), and more coexisting HT (13.4% vs. 30.3%; *p* < 0.001) than female patients did (Table 1). No patients had distant metastases. There were no significant differences between sexes in terms of other clinicopathological factors, including age, multifocality, and margin involvement. Seven (4.5%) male patients and 20 (1.8%) female patients experienced recurrence (*p* = 0.013). Log-rank analysis also demonstrated that the five-year DFS rate was significantly lower among males (94.9% vs. 98.5%; *p* = 0.029) than among females (Figure 1A).

### 3.2. Comparison of Disease-Free Survival in the Matched Cohorts

The clinicopathological characteristics after matching are summarized in Table 2. The matched cohorts did not differ in terms of tumor size, ETE, LN metastasis, and coexisting HT. The overall recurrence rate showed no statistical difference between the male and female patients (4.5% vs. 3.2%; *p* = 0.452) after adjusting for potential confounders. The five-year DFS rates among males and females were also comparable (94.9% vs. 96.9%; *p* = 0.616) (Figure 1B).

### 3.3. Predictors of Poor DFS in Patients with PTC

Univariable Cox proportional hazards regression analysis suggested that male sex (HR 2.526, 95% CI 1.068–5.976), tumor size (HR 1.841, 95% CI 1.319–2.567), microscopic ETE (HR 2.894, 95% CI 1.096–7.644), multifocality (HR 2.390, 95% CI 1.109–5.151), and N1b LN metastasis (HR 7.938, 95% CI 3.061–20.57) significantly increased the risk of recurrence (Table 3). Only N1b LN metastasis (HR 4.204 95% CI 1.483–11.92) retained statistical significance in the multivariable analysis, while male sex (HR 1.982, 95% CI 0.831–4.726) was not a significant predictor.

### 3.4. Subgroup Analysis of the Impact of Patient Sex with Adjustments for Possible Confounders

**Figure 2:**
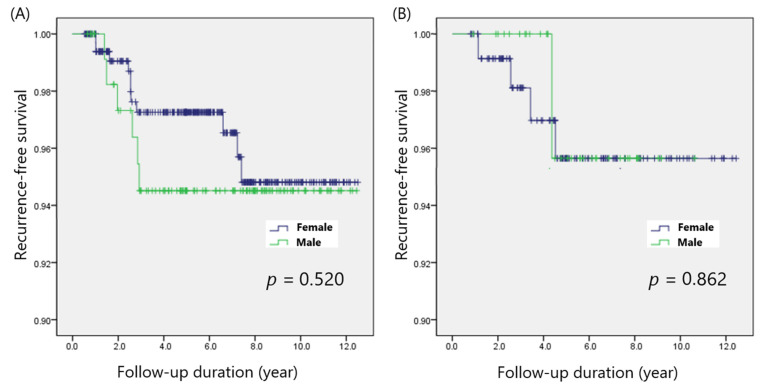
Disease-free survival in patients with (**A**) age < 55 years and (**B**) age ≥ 55 years.

There were 469 patients <55 years of age, and 159 ≥55 years old. Among patients <55 years old, recurrences were observed in 6 (5.0%) of 121 male patients and 11 (3.2%) of 348 female patients (*p* = 0.362). Among patients ≥55 years old, 1 (2.8%) of 36 male patients and 4 (3.3%) of 123 female patients developed recurrences (*p* = 0.886). Kaplan–Meier survival plots also demonstrated that male patients showed five-year DFS rates comparable to those of female patients in both the <55-year (94.5% vs. 97.3%; *p* = 0.520) and ≥55-year age groups (95.7% vs. 95.6%; *p* = 0.862) (Figure 2).

**Figure 3:**
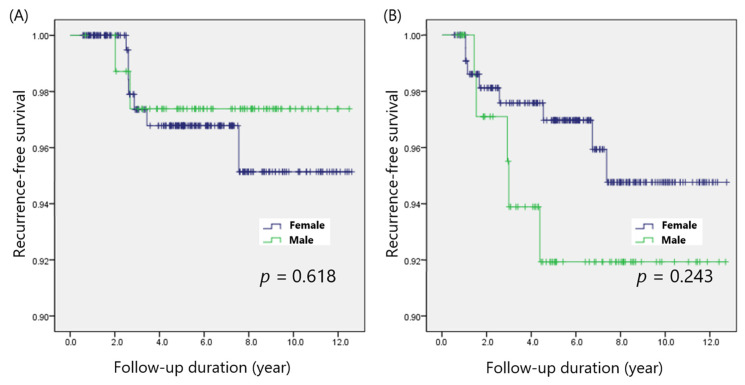
Disease-free survival in patients with (**A**) PTMC and (**B**) non-PTMC.

Papillary thyroid microcarcinoma (PTMC; defined as PTC ≤ 1 cm) was found in 325 patients, and 303 patients had PTC > 1 cm (non-PTMC). The recurrence rates among male and female patients were 2 of 80 (2.5%) and 7 of 245 (2.9%; *p* = 0.866), respectively, in the PTMC group, and 5 of 77 (6.5%) and 8 of 226 (3.5%; *p* = 0.269), respectively, in the non-PTMC group. The five-year DFS was not significantly different between male and female patients in both the PTMC (97.4% vs. 96.8%; *p* = 0.618) and non-PTMC (91.9% vs. 97.0%; *p* = 0.243) groups (Figure 3).

## 4. Discussion

The present study demonstrated that male sex was not associated with the risk of recurrence, although male patients with PTC tended to have aggressive clinicopathological features, including larger tumor size, ETE, and LN metastasis. As male sex is associated with risk factors for recurrence, such as tumor size, ETE, lymphovascular invasion, LN metastasis, and extranodal extension, some researchers have wondered whether male sex itself increases the risk of recurrence [10,16,18]. In the present study, therefore, we performed propensity score matching to minimize the effects of confounding factors. After adjusting for potential confounders, five-year DFS rates were comparable between male and female patients (94.9% vs. 96.9%; *p* = 0.616), and we confirmed that male sex was not an independent risk factor for recurrence in patients with PTC.

In the present study, we defined recurrence as the occurrence of a disease event when the disease-free condition persisted for at least one year after initial surgery, according to the latest American Thyroid Association guidelines [19]. Patients with persistent disease, which was defined as structural disease occurring within the first year after surgery, were excluded from our study. Persistent disease can be associated with worse outcomes compared with recurrent disease; however, most previous studies did not evaluate persistent or recurrent PTC independently [20,21]. Sapuppo et al. demonstrated that male sex was not associated with an increased risk of recurrence but that it was, instead, associated with persistent disease [22]. Therefore, we only included true recurrences without persistent disease and concluded that male sex was not associated with recurrence.

The effect of sex could vary according to tumor size or patient age. Lee et al. indicated that male sex was not an independent prognostic factor for recurrence in PTMC, but it was an independent prognostic factor in the context of PTC > 1 cm [23]. Jonklaas et al. reported that women <55 years old had improved disease-specific survival compared with men, while both men and women >55 years had similar outcomes [24]. Oyer et al. also demonstrated that male sex was a risk factor for recurrence in patients younger than 45 years [25]. In contrast, Park et al. showed that male sex was not an independent risk factor for recurrence regardless of patient age after propensity score matching [16]. In the present study, we also confirmed that the impact of sex was not associated with tumor size or patient age when possible confounders were adjusted for.

N1b LN metastasis significantly increased the risk of recurrence in the present study. Previous studies have indicated N1b LN metastasis to be one of the most effective markers of poor prognosis among thyroid cancer patients with recurrent disease and persistent disease [22,26,27,28]. N1b LN metastasis further has been recognized as a predictor of both distant metastasis and cancer-related death. As male patients have a higher risk of N1b LN metastasis, men have tended to undergo LN dissection and receive radioactive iodine more frequently than women [10]. Nilubol et al. also found that more aggressive treatment was performed in men than in women in their study of men and women with thyroid cancer [29]. This more aggressive treatment could lower the risk of recurrence, resulting in comparable recurrence-free survival rates between male and female patients. Our results were consistent with those previous reports.

This study had some limitations. First, this was a retrospective cohort study, which might be prone to selection bias. For example, the selection of patients who underwent radioiodine ablation could have been influenced by various factors. Second, we did not include information on the PTC mutational profiles, including information about BRAF mutations. BRAF mutations can increase the risk of recurrence, and the effects of BRAF mutations can vary according to sex [30]. Additional studies on the impact of sex on PTC, with additional BRAF mutation analysis, are warranted. Finally, we did not evaluate long-term prognosis, such as mortality. The follow-up period of 6.6 years was not sufficient to assess cancer-specific survival.

## 5. Conclusions

Male sex was not associated with the risk of recurrence in patients with PTC. Male patients do not always require aggressive treatment and follow-up approaches. Further studies with mutation profile analyses and a longer follow-up period should be conducted to clarify the impact of sex on the prognosis of PTC.

## Figures and Tables

**Table 1:** Comparison of clinicopathological characteristics between male and female patients with papillary thyroid carcinoma.

Characteristics	Male (*n* = 157)	Female (*n* = 1095)	*p*-Value
Age (years)	46.7 ± 11.2	47.6 ± 11.3	0.322
Pathologic characteristics			
Tumor size (cm)	1.3 ± 0.9	1.0 ± 0.6	<0.001
Extrathyroidal extension			0.018
No	56 (35.7%)	425 (38.8%)	
Microscopic	98 (62.4%)	667 (60.9%)	
Gross	3 (1.9%)	3 (0.3%)	
Multifocality	64 (40.8%)	428 (39.1%)	0.687
LN metastasis			0.001
N0	73 (46.5%)	650 (59.4%)	
N1a	58 (36.9%)	350 (32.0%)	
N1b	26 (16.6%)	95 (8.7%)	
Margin involvement	5 (3.2%)	38 (3.5%)	0.854
Coexisting HT	21 (13.4%)	332 (30.3%)	<0.001
Postoperative management			
^131^I remnant ablation	77 (49.0%)	466 (42.6%)	0.125
^131^I dose (mCi)	144.1 ± 33.1	132.5 ± 35.4	0.006
Follow-up period (years)	6.2 ± 3.0	6.6 ± 3.2	0.078
Recurrence	7 (4.5%)	20 (1.8%)	0.034

LN, lymph node; HT, Hashimoto thyroiditis.

**Table 2:** Comparison of clinicopathological characteristics according to sex after 1:3 propensity score matching.

Characteristics	Male (*n* = 157)	Female (*n* = 471)	*p*-Value
Age (years)	46.7 ± 11.2	47.6 ± 12.0	0.397
Pathologic characteristics			
Tumor size (cm)	1.3 ± 0.9	1.2 ± 0.8	0.258
Extrathyroidal extension			0.134
No	56 (35.7%)	153 (32.5%)	
Microscopic	98 (62.4%)	316 (67.1%)	
Gross	3 (1.9%)	2 (0.4%)	
Multifocality	64 (40.8%)	197 (41.8%)	0.815
LN metastasis			0.503
N0	73 (46.5%)	200 (42.5%)	
N1a	58 (36.9%)	199 (42.3%)	
N1b	26 (16.6%)	72 (15.3%)	
Margin involvement	5 (3.2%)	22 (4.7%)	0.427
Coexisting HT	21 (13.4%)	58 (12.3%)	0.728
Postoperative management			
^131^I remnant ablation	77 (49.0%)	263 (55.8%)	0.139
^131^I dose (mCi)	144.1 ± 33.1	135.4 ± 35.0	0.054
Follow-up period (years)	6.2 ± 3.0	5.6 ± 3.1	0.037
Recurrence	7 (4.5%)	15 (3.2%)	0.452

PTC, papillary thyroid carcinoma; ETE, extrathyroidal extension; LN, lymph node; HT, Hashimoto thyroiditis.

**Table 3:** Cox proportional hazards analysis for predictive factors of PTC recurrence.

Characteristics	Univariable Analysis		Multivariable Analysis	
	HR (95% CI)	*p*-Value	HR (95% CI)	*p*-Value
Age (years)	0.987 (0.952–1.023)	0.470		
Male sex	2.526 (1.068–5.976)	0.035	1.982 (0.831–4.726)	0.123
Tumor size (cm)	1.841 (1.319–2.567)	<0.001	1.504 (0.995–2.275)	0.053
Extrathyroidal extension				
Microscopic	2.894 (1.096–7.644)	0.032	1.858 (0.682–5.060)	0.226
Gross	<0.01 (0.0–infinite)	0.985	0.000 (0.0–infinite)	0.979
Multifocality	2.390 (1.109–5.151)	0.026	2.105 (0.972-4.558)	0.059
LN metastasis				
N1a	2.166 (0.855–5.490)	0.103	1.642 (0.640–4.213)	0.302
N1b	7.938 (3.061–20.57)	<0.001	4.204 (1.483–11.92)	0.007
Margin involvement	2.860 (0.677–12.08)	0.153	2.062 (0.475–8.953)	0.334
Hashimoto thyroiditis	0.602 (0.228–1.589)	0.305		

HR, hazard ratio; CI, confidence interval; LN, lymph node.

## Data Availability

The data presented in this study are available upon request from the corresponding author. The data are not publicly available due to institutional policy.
